# Whole-Genome Analysis of *Halomonas* sp. H5 Revealed Multiple Functional Genes Relevant to Tomato Growth Promotion, Plant Salt Tolerance, and Rhizosphere Soil Microecology Regulation

**DOI:** 10.3390/microorganisms13081781

**Published:** 2025-07-30

**Authors:** Yan Li, Meiying Gu, Wanli Xu, Jing Zhu, Min Chu, Qiyong Tang, Yuanyang Yi, Lijuan Zhang, Pan Li, Yunshu Zhang, Osman Ghenijan, Zhidong Zhang, Ning Li

**Affiliations:** 1 College of Resource and Environment, Xinjiang Agricultural University, Urumqi 830052, China; liyansuho0805@sina.com; 2 Xinjiang Laboratory of Special Environmental Microbiology, Institute of Microbiology, Xinjiang Uygur Autonomous Region Academy of Agricultural Sciences, Urumqi 830091, China; gmyxj2008@163.com (M.G.); zhujing2020@hotmail.com (J.Z.); chuliu2002@163.com (M.C.); tqy25@163.com (Q.T.); 15022970169@163.com (Y.Y.); zhanglijuan@xaas.ac.cn (L.Z.); ghenijan_osman@126.com (O.G.); 3 Institute of Agricultural Resources and Environment, Xinjiang Uygur Autonomous Region Academy of Agricultural Sciences, Urumqi 830091, China; wlxu2005@163.com (W.X.); lipanxj@sina.com (P.L.); zhangxl1994@sina.com (Y.Z.); 4 Key Laboratory of Saline-Alkali Soil Improvement and Utilization (Saline-Alkali Land in Arid and Semi-Arid Regions), Ministry of Agriculture and Rural Affairs, Urumqi 830091, China

**Keywords:** glycophyte plants, microbial inoculant, bacterial genes, plant physiology, rhizosphere soil microbiome, plant–microbial interaction

## Abstract

Soil salinity adversely affects crop growth and development, leading to reduced soil fertility and agricultural productivity. The indigenous salt-tolerant plant growth-promoting rhizobacteria (PGPR), as a sustainable microbial resource, do not only promote growth and alleviate salt stress, but also improve the soil microecology of crops. The strain H5 isolated from saline-alkali soil in Bachu of Xinjiang was studied through whole-genome analysis, functional annotation, and plant growth-promoting, salt-tolerant trait gene analysis. Phylogenetic tree analysis and 16S rDNA sequencing confirmed its classification within the genus *Halomonas*. Functional annotation revealed that the H5 genome harbored multiple functional gene clusters associated with plant growth promotion and salt tolerance, which were critically involved in key biological processes such as bacterial survival, nutrient acquisition, environmental adaptation, and plant growth promotion. The pot experiment under moderate salt stress demonstrated that seed inoculation with *Halomonas* sp. H5 not only significantly improved the agronomic traits of tomato seedlings, but also increased plant antioxidant enzyme activities under salt stress. Additionally, soil analysis revealed H5 treatment significantly decreased the total salt (9.33%) and electrical conductivity (8.09%), while significantly improving organic matter content (11.19%) and total nitrogen content (10.81%), respectively (*p* < 0.05). Inoculation of strain H5 induced taxonomic and functional shifts in the rhizosphere microbial community, increasing the relative abundance of microorganisms associated with plant growth-promoting and carbon and nitrogen cycles, and reduced the relative abundance of the genera *Alternaria* (15.14%) and *Fusarium* (9.76%), which are closely related to tomato diseases (*p* < 0.05). Overall, this strain exhibits significant potential in alleviating abiotic stress, enhancing growth, improving disease resistance, and optimizing soil microecological conditions in tomato plants. These results provide a valuable microbial resource for saline soil remediation and utilization.

## 1. Introduction

Soil salinization has emerged as a global environmental challenge that severely limits crop productivity and impedes sustainable agricultural development. Notably, projections indicate that by 2050, nearly half of the arable land will be impacted by the continuous accumulation of salts, salty irrigation, and climate change [1]. This phenomenon is expected to impair salt-sensitive crops growth, disrupt physiological processes, and damage soil ecosystems, while simultaneously threatening global food security, agricultural productivity, and ecosystem health.

Salt stress is a major abiotic stressor that affects the growth and development of plants (both glycophytes and halophytes) through multifaceted mechanisms under high-salinity conditions. Glycophytes, which comprise the majority of plant species, including most crops, exhibit high sensitivity to salt stress. Increased salt concentrations induce osmotic imbalance, ionic toxicity, alkaline stress, oxidative damage, and water deficit in glycophytes, which collectively impede water and nutrient absorption, inhibit DNA replication and macromolecule biosynthesis, and negatively impact seed germination, growth, physiological processes, and biochemical pathways [2,3]. Furthermore, persistent salt accumulation leads to a progressive deterioration of soil ecosystem functions through alterations in biogeochemical cycling and microbial community dynamics [4]. In contrast, halophytes exhibit the remarkable ability to sustain metabolic activity under high-salinity conditions. They possess a greater number of gene copies associated with osmosensing, signal transduction, and stress adaptation mechanisms. Morphologically, halophytes are characterized by specialized structures, such as salt glands or bladders, which facilitate active salt excretion. Additionally, their succulent tissues enable ion dilution, while selective ion uptake and compartmentalization strategies further enhance their salt tolerance. Notably, certain euhalophytes actually require moderate salinity levels to achieve optimal growth, highlighting a unique adaptation that differentiates them from glycophytes [5].

The remediation of saline soils for the cultivation of glycophytic crops involves a multifaceted approach, encompassing physical, chemical, and biological strategies. While certain conventional remediation techniques have demonstrated efficacy in salinity mitigation, their practical implementation is often constrained by substantial operational costs and potential adverse impacts on critical soil properties, such as structural integrity, nutrient cycling capacity, and microbial community dynamics. Microbial remediation has emerged as a promising strategy for improving salt-affected soils [6]. This approach can reduce dependence on agrochemicals during plant growth and metabolic processes while safeguarding food security. Owing to its advantages of high efficiency, low cost, environmental safety, and long-term sustainability, this technology has garnered considerable attention in soil salinity remediation research [7].

Plant growth-promoting bacteria, which successfully colonize the rhizosphere soil of plants, represent a valuable microbial resource with significant agricultural applications in saline soil remediation [8]. They can effectively alleviate plant physiological damage caused by salt stress, enhance mineral nutrient uptake by plants, and exert antagonistic effects against pathogenic bacteria. As a globally important glycophytic crop, tomato (*Solanum lycopersicum* L.) provides essential nutritional components while exhibiting particular sensitivity to saline conditions. Progressive soil salinization has led to growth inhibition, yield reduction, and fruit quality compromise.

Currently, the salt-tolerant plant growth-promoting strains widely applied in tomato cultivation primarily include *Bacillus*, *Enterobacter*, *Pantoea*, *Lactobacillus*, *Serratia*, *Pseudomonas*, *Staphylococcus*, *Agrobacterium, and Streptomyces*. Furthermore, microorganisms such as arbuscular mycorrhizal fungi, *Aspergillus,* and *Trichoderma* also play significant roles in promoting salt tolerance and growth in tomato plants [9,10,11,12,13,14,15,16,17,18,19]. Under saline-stressed environments, PGPR stimulate plant metabolic processes to promote phytohormone biosynthesis [20], trigger antioxidant defense systems [21], induce siderophore production [22], and enhance nutrient absorption and photosynthesis [23]. Furthermore, these bacteria synthesize plant hormones such as indole-3-acetic acid (IAA) and cytokinins [24]. In addition, ACC deaminase is also a very important characteristic of PGPR, as it can stimulate plant growth in salt stress conditions by reducing the level of the stress hormone ethylene [25]. Through pathogen antagonism, mineral dissolution, and nitrogen fixation, they promote the absorption of nutrients by plant roots, improve soil health, and inhibit soil-borne pathogens [26]. Soil microbial community diversity plays a crucial role in enhancing nutrient availability and soil health. Research indicates that PGPR colonization in the rhizosphere selectively modulates microbial abundance, by altering dominant populations, thereby optimizing microbiome structure. This ecological restructuring enhances soil fertility, plant stress resistance, and adaptive capacity under salt stress [27,28].

The diverse saline-alkali ecosystems of Xinjiang harbor abundant microbial resources. Selecting indigenous PGPR adapted to local crops and environmental conditions is critical. This study conducted whole-genome sequencing of a previously isolated *Halomonas* sp. H5 and evaluated its efficacy in alleviating salt stress and enhancing tomato growth. Subsequent investigations combining soil physicochemical property analysis and high-throughput sequencing technology were employed to assess the impact of a salt-tolerant PGPR inoculant on rhizosphere soil microecological health. These findings provide valuable microbial resources for the development of biofertilizers and establish a theoretical foundation for improving soil microenvironments under salt stress.

## 2. Materials and Methods

### 2.1. Experimental Materials

The salt-tolerant and plant growth-promoting strain *Halomonas* sp. H5 was isolated from saline-alkali soil in Bachu County, Xinjiang Uygur Autonomous Region, China (78.64 °E, 39.79 °N), using the dilution-coated plate isolation method [29]. Aliquots (100 μL) from appropriate dilutions were spread-plated onto nutrient agar medium (beef extract 3.0 g/L, peptone 10.0 g/L, NaCl 5.0 g/L, and agar 20.0 g/L; pH 7.3 ± 0.1) containing 5% NaCl and incubated at 30 °C for 72 h. After colony formation, distinct colonies were subcultured onto fresh media to obtain pure isolates. These isolates were subsequently preserved by a vacuum freeze-drying method and deposited in the Microbiological Culture Collection Center of Xinjiang, Xinjiang Academy of Agricultural Sciences (XJAAS), China.

The soil used in this pot experiment was collected from moderately saline-alkaline soil (total salt content of 5.66 g/kg, pH 8.34) in Bachu County. The basic physicochemical properties of the pot experiment soil were as follows: pH 8.34, total salt content of 5.66 g/kg, electrical conductivity of 1.42 mS/cm, organic matter content of 7.00 g/kg, total nitrogen content of 0.43 g/kg, available nitrogen concentration of 58.21 mg/kg, available phosphorus concentration of 15.3 mg/kg, and available potassium concentration of 155 mg/kg. During soil collection, the topsoil, litter, residual film, gravel, and weeds were removed. Soil from the 0–20 cm cultivated layer was excavated, mixed thoroughly, and stored for subsequent experimental use.

### 2.2. Experimental Methods

#### 2.2.1. Whole-Genome Sequencing and Bioinformatics Analysis of *Halomonas* sp. H5

*Halomonas* sp. H5 was cultured on nutrient agar (NA) plates containing 2% NaCl at 30 °C for 48 h. After the colonies had grown, the 16S rRNA gene was amplified using primers 27F/1492R [30]. The obtained 16S rRNA gene sequences were compared with those in the NCBI GenBank database (http://www.ncbi.nlm.nih.gov accessed on 6 July 2025) using BLASTn 1.4.0 for taxonomic identification. The phylogenetic tree of *Halomonas* was constructed using the neighbor-joining method in MEGA 7.0 software.

Whole-genome sequencing was performed by Novogene Bioinformatics Technology Co., Ltd. (Beijing, China). Genomic DNA was extracted using the sodium-Tris-EDTA (STE) lysis method, followed by quality verification through 0.8% agarose gel electrophoresis. The qualified DNA sample was sheared into fragments of optimal size for library construction using a Covaris g-TUBE device (Covaris, Woburn, MA, USA). A SMRTbell™ library was constructed using the SMRT bell™ Template Kit 2.0 (Pacific Biosciences, Menlo Park, CA, USA) and subsequently sequenced on the PacBio platform.

The genome assembly was performed using Canu 2.0 software. The assembled contigs were subjected to three rounds of error correction using Racon 1.4.13, leveraging third-generation sequencing data. Subsequently, three rounds of error correction using Pilon 1.22 with second-generation sequencing reads were conducted to obtain the optimal contig sequences. The contigs were then linked using paired-end or mate-pair information. Local assembly and optimization were performed based on paired-end reads and overlap relationships to generate scaffolds [31].

#### 2.2.2. Salt Tolerance Test of Tomato Seedlings Treated with *Halomonas* sp. H5

*Halomonas* sp. H5 was cultured in nutrient broth medium (beef extract 3.0 g/L, peptone 10.0 g/L, and NaCl 5.0 g/L; pH 7.3 ± 0.1) containing 2% NaCl at 30 °C and 150 rpm for 48 h. Cells were collected by centrifugation at 5590× *g* for 10 min, and the pellet was resuspended in sterile deionized water to an optical density at 600 nm (OD_600_) of 1 × 10^8^ CFU (colony forming unit)/mL for subsequent experiments. Tomato seeds were sterilized in 1% sodium hypochlorite aqueous solution for 2 min. The seeds were further washed three times with distilled water [32]. Sterilized tomato seeds were soaked in sterile 2% (*v*/*v*) *Halomonas* sp. H5 cell suspension (H5 treatment) for 4 h, while control-group seeds were treated with an equivalent volume of sterile deionized water under identical conditions (CK treatment). Pre-soaked tomato seeds were transferred to agar plates containing 0.5% (*v*/*v*) NaCl and cultured at room temperature [33]. Seedlings’ salt tolerance was evaluated following 14 days of salt exposure.

#### 2.2.3. Design of the Pot-Based Tomato Cultivation Experiment

Moderately saline-alkaline soil (350 g dry weight per pot) was placed into plastic pots (10 cm in diameter × 7.5 cm in height) and prepared for the experiment. Tomato seeds pre-treated with CK treatment and H5 treatment (prepared as described in Section 2.2.2) were sown in each pot at a density of 10 seeds per pot, with a sowing depth of approximately 1 cm. The experiment included three replicates per treatment group, and plants were cultivated in a greenhouse maintained at 25 °C for a period of 2 months [34]. Tomato plants in the CK treatment and H5 treatment groups were maintained under identical irrigation conditions, with each pot receiving 50 mL of distilled water every 2 days to ensure consistent soil water content (mean ± 0.5% variation between treatments).

#### 2.2.4. Measurement of Growth and Physiological Parameters in Tomato Plants

Seedling emergence rates were recorded for the H5 treatment and CK treatment. Plant height and stem diameter were measured using a ruler, and fresh weight was determined with an electronic balance. Leaf physiological indices, including chlorophyll content, malondialdehyde (MDA) content, and proline (PRO) content, were analyzed. Antioxidant enzyme activities of superoxide dismutase (SOD), catalase (CAT), and polyphenol oxidase (PPO) were assayed. All parameters were quantified via colorimetric methods using commercial assay kits (Griess Biotechnology Co., Ltd., Suzhou, China).

#### 2.2.5. Determination of Physicochemical Properties of Rhizosphere Soil

Rhizosphere soil samples were collected using a root-shaking method. Soil particles adhering to excavated tomato roots were gently shaken off and retained as rhizosphere soil [35]. Rhizosphere soil from each pot was homogenized and divided into two portions. One portion was stored at −80 °C for microbial community analysis by high-throughput sequencing. The other was air-dried, passed through a 2 mm sieve, and used for the determination of soil physicochemical properties

Soil properties were analyzed according to Analytical Methods for Soil and Agricultural Chemistry [36]. The pH was measured potentiometrically with a calibrated pH meter. Total salt content was measured by gravimetric analysis after oven-drying at 105 °C. Electrical conductivity (EC) was measured by a digital conductivity meter. Available nitrogen (AN) was measured by the alkali solution diffusion method. Available phosphorus (AP) was measured by the sodium bicarbonate extraction–molybdenum antimony anti-colorimetric method. Available potassium (AK) was measured by the ammonium acetate extraction–flame photometry method. Soil organic matter (OM) was measured by the potassium dichromate method. Total nitrogen (TN) was measured by the Kjeldahl digestion method.

#### 2.2.6. Determination of Microbial Community Structure and Diversity of the Rhizosphere Soils

Soil genomic DNA was extracted using the FastDNA^®^ SPIN Kit for Soil (MP Biomedicals, Irvine, CA, USA). The purity and concentration of extracted DNA were subsequently assessed through electrophoretic analysis on 0.8% (*w*/*v*) agarose gels. PCR amplification of the extracted soil DNA was performed using specific primers. For bacteria communities, the V3-V4 hypervariable region of the 16S rRNA gene was amplified with primers 338F (5′-ACTCCTACGGGAGGCAGCAG-3′) and 806R (5′-GGACTACHVGGGT WTCTAAT-3′). For fungal communities, the ITS1-ITS2 region was amplified using the primers ITS1F (5′-CTTGGTCATTTAGAGGAAGTAA-3′) and ITS2R (5′-GCTGCGTTCTT CATCGATGC-3′) [37].

Following purification of the amplification products, library construction and sequencing were conducted on the Illumina HiSeq 2500 platform by Novogene Bioinformatics Technology Co., Ltd. (Beijing, China). Raw sequencing data underwent assembly and quality filtering to generate high-quality clean reads. Denoising analysis was subsequently performed on the clean data using DADA2 to obtain exact amplicon sequence variants (ASVs).

### 2.3. Data Analysis

For the assembled genome sequence of *Halomonas* sp. H5, the predicted coding genes were combined and visualized using the Circos v0.69-9 software. The whole-genome protein sequences of *Halomonas* sp. H5 were aligned against functional databases including Clusters of Orthologous Groups of proteins (COG), Gene Ontology (GO), Kyoto Encyclopedia of Genes and Genomes (KEGG), and the Carbohydrate-Active enZYmes Database (CAZy) using diamond with a stringent e-value threshold of ≤1 × 10^−5^. The highest-scoring hits meeting default parameters (sequence identity ≥ 40% and coverage ≥ 40%) were subsequently selected for comprehensive bioinformatics functional annotation and analysis. The secondary metabolite gene clusters in the genome were predicted using the antiSMASH v4.0.2 program. The virulence factor genes were annotated using the VFDB (Virulence Factors of Pathogenic Bacteria) databases.

The raw sequencing data obtained from high-throughput sequencing were assembled and filtered to generate high-quality target sequences (ASVs) suitable for downstream analyses. Representative sequences of ASVs were taxonomically annotated using bacterial and fungal reference databases to determine their phylogenetic classifications, abundance profiles, and distribution patterns. All subsequent bioinformatics analyses were performed using QIIME2 v2.5. Alpha diversity metrics were calculated for the ASV dataset, with data visualization conducted through boxplot generation using the Chiplot v1.1.0 online platform (https://www.chiplot.online accessed on 24 June 2025). Functional prediction analyses for bacterial and fungal communities were conducted using FAPROTAX v1.2.4 and FunGuild v1.2 software, respectively. Statistical analyses and scientific visualizations were primarily implemented using R v4.3.1, Python v3.10, and Java v17.

Statistical analyses were performed using SPSS 19.0 software (IBM Corp., Armonk, NY, USA). A one-way analysis of variance (ANOVA) was used to assess significant differences among treatments (*p* < 0.05).

## 3. Results

### 3.1. Analysis of Whole-Genome Sequencing and Functional Genome of Halomonas sp. H5

#### 3.1.1. Analysis of Whole-Genome Sequencing and Composition of *Halomonas* sp. H5

**Figure 1:**
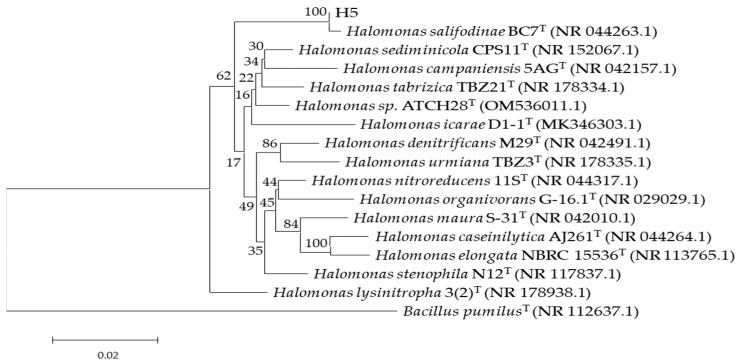
Phylogenetic tree based on 16S rRNA gene sequences.

As shown in Figure 1, the phylogenetic analysis of the 16S rRNA gene sequence, conducted using the NCBI database, revealed that strain H5 belongs to the genus *Halomonas,* exhibiting 99.93% sequence identity within the cluster of the species *Halomonas salifodinae*.

**Figure 2:**
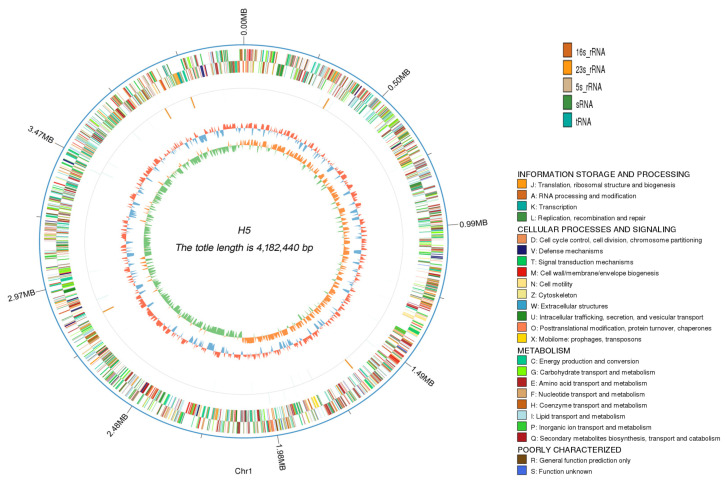
Genomic circle map of *Halomonas* sp. H5. The concentric circles, arranged from the outermost to the innermost, represent distinct genomic features: genomic sequence coordinates, gene functional annotations, non-coding RNA distributions, genomic GC content profiles, and GC skew patterns. In the GC content ring, inward-directed blue segments denote regions with below-average GC content (relative to the genome-wide mean), whereas outward-protruding red segments indicate above-average GC regions. The innermost GC skew ring features green segments oriented toward the center, representing genomic regions where guanine (G) content is lower than cytosine (C); correspondingly complementary orange segments extending outward indicate the opposite trend (G > C).

Previous studies have demonstrated that *Halomonas* sp. H5 exhibits significant plant growth-promoting (PGP) traits under high-salinity conditions. To elucidate the molecular mechanisms underlying its salt tolerance and PGP capacities, we performed whole genome sequencing. A circular genome map was generated by integrating the genome assembly and scaffolding results with predicted protein-coding gene annotations (Figure 2). Genomic analysis revealed that *Halomonas* sp. H5 possesses a circular chromosome of 4,182,440 bp with a G + C content of 66.81%. The genome contained 3774 protein-coding genes spanning 3,626,841 bp, averaging 961 bp in length. It included 66 tRNAs, five copies each of 5S rRNA, 16S rRNA, and 23S rRNA, along with three sRNAs. Additionally, seven genomic islands (GIs), prophages, and three CRISPR arrays were identified.

#### 3.1.2. Analysis of Functional Genome Annotation

The protein sequences of *Halomonas* sp. H5 were systematically annotated by comparing them against multiple functional databases. The analysis revealed the following distribution of annotated genes across databases: NR (3637, 96.37%), Swiss-Prot (1929, 51.11%), KEGG (3543, 93.88%), COG (3092, 81.93%), TCDB (459, 12.16%), GO (2471, 65.47%), CAZy (167, 4.43%), Pfam (2471, 65.47%), PHI (585, 15.50%), VFDB (295, 7.82%), and CARD (1, 0.03%). Percentages indicate the proportion of annotated genes relative to the total gene count (3773 genes).

##### Analysis of COG Annotation Results

**Figure 3:**
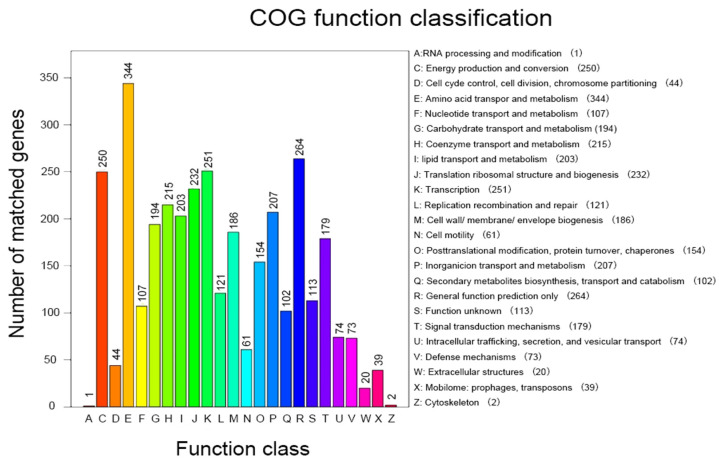
COG functional classification of annotated genes in *Halomonas* sp. H5.

Functional genome annotation of *Halomonas* sp. H5 based on the COG database (Figure 3) revealed significant gene enrichment in categories associated with plant growth-promoting mechanisms. The most predominant functional categories were amino acid transport and metabolism (344 genes, 10.01%), transcription (251 genes, 7.31%), and energy production and conversion (250 genes, 7.28%). Subsequent analysis identified additional categories with proportions ≥5%, including translation, ribosomal structure, and biogenesis (232 genes, 6.75%); coenzyme transport and metabolism (215 genes, 6.26%); inorganic ion transport and metabolism (207 genes, 5.91%); lipid transport and metabolism (203 genes, 5.65%); carbohydrate transport and metabolism (194 genes, 5.41%); cell wall/membrane/envelope biogenesis (186 genes, 5.91%); and signal transduction mechanisms (179 genes, 5.21%). Additionally, secondary metabolite biosynthesis, transport, and catabolism (102 genes, 2.97%) was closely related to plant growth-promoting mechanisms.

##### Analysis of GO Annotation Results

**Figure 4:**
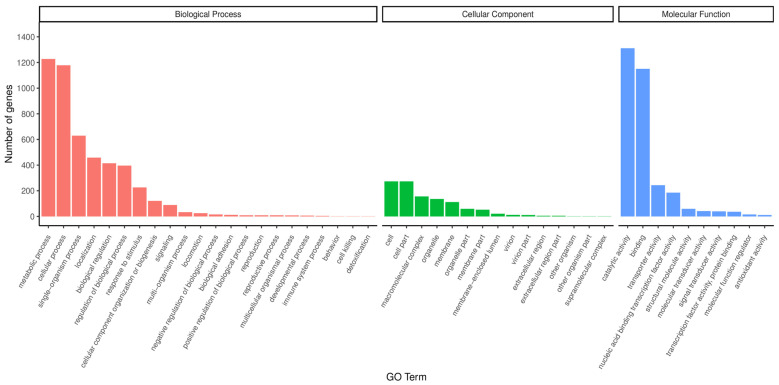
GO functional classification of annotated genes in *Halomonas* sp. H5.

Functional analysis of the GO database (Figure 4) revealed that strain H5 exhibited enrichment of 4871, 3090, and 1118 genes in the Biological Process, Molecular Function, and Cellular Component categories, respectively. Within the Biological Process category, predominant annotations included metabolic process (1227 genes) and cellular process (1178 genes). For Molecular Function, annotations were primarily associated with catalytic activity (1311 genes) and binding functions involving DNA, ATP, and metal ions (1150 genes). In the Cellular Component analysis, the cell and cell part subcategories each contained 273 annotated genes. Genes related to growth promotion and stress resistance encompassed those associated with metabolic processes (1227 genes), catalytic activity (1311 genes), nucleic acid-binding transcription factor activity (185 genes), transcription factor activity and protein binding (36 genes), transporter activity (243 genes), and antioxidant activity (11 genes).

##### Analysis of KEGG Annotation Results

**Figure 5:**
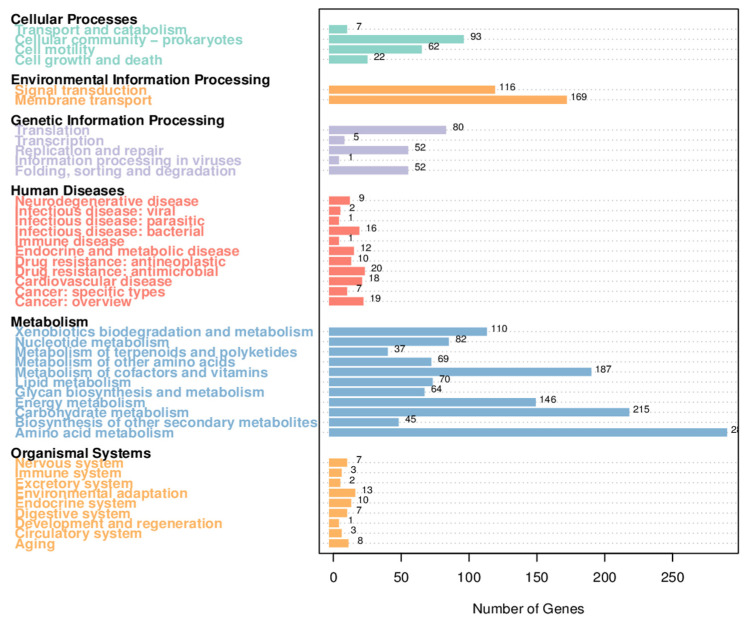
Functional annotation of genes based on KEGG metabolic pathway classification in *Halomonas* sp. H5.

Bioinformatic analysis of the H5 genome using the KEGG v112.0 database revealed distinct functional characteristics (Figure 5). Among the 3543 annotated genes, metabolic pathway-related genes represented the most abundant functional category, comprising.

1635 genes (46.15%). A substantial proportion of these metabolic genes exhibited significant associations with critical plant physiological processes, particularly those involved in stress resistance mechanisms and growth promotion. Functional annotation of genes highlighted distinct distribution patterns across metabolic pathways. Specifically, amino acid metabolism constituted the largest category (451 genes), followed by carbohydrate metabolism (323 genes). Genes associated with cofactor and vitamin metabolism, as well as xenobiotics biodegradation and metabolism, each accounted for 193 genes. Energy metabolism-related genes (including carbon fixation in photosynthetic organisms, prokaryotic carbon fixation pathways, methane metabolism, nitrogen metabolism, and photosynthesis) totaled 166 genes. In addition, there were a small number of genes related to secondary metabolite biosynthesis (49 genes), terpenoid and polyketide metabolism (45 genes), and polysaccharide synthesis/metabolism (42 genes). Moreover, strain H5 harbored 33 genes related to arginine and proline metabolism, 58 genes associated with glycine, serine, and threonine metabolism, and 10 genes within ectoine secondary metabolism clusters. These genetic features collectively demonstrate sophisticated molecular strategies for halotolerance through compatible solute accumulation.

The metabolic pathways associated with osmotic regulation included bacterial chemotaxis (25 genes) and flagellar assembly (46 genes) under cell motility, biofilm formation (55 genes) and quorum sensing (55 genes) under cellular community-prokaryotes, and peroxisome-related functions (6 genes) within transport and catabolism pathways. In environmental information processing, membrane transport pathways contained 135 genes annotated as ABC transporters. Signal transduction pathways encompassed 100 genes related to two-component systems, 6 to phosphatidylinositol signaling, 4 to HIF-1 signaling, and 3 to plant MAPK signaling. Additionally, the genome of *Halomonas* sp. H5 harbors 25 siderophore biosynthesis gene clusters and 24 genes involved in tryptophan synthesis, a precursor of IAA. Antioxidant defense systems were notably represented, with 6 peroxidase genes, 16 glutathione metabolism-related genes, and 4 genes participating in ascorbate/aldarate metabolism pathways.

##### Analysis of CAZy Annotation Results

**Figure 6:**
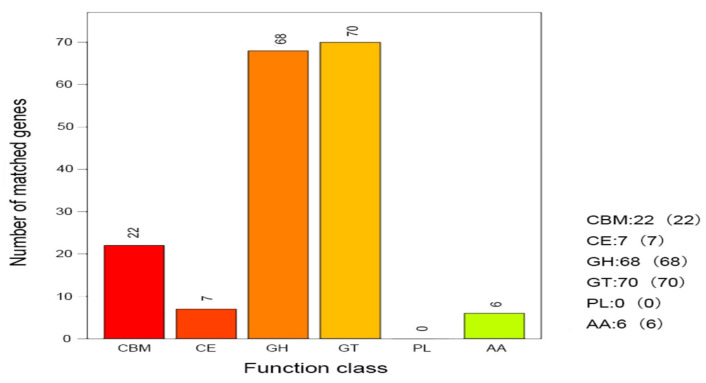
Functional classification and genomic distribution of carbohydrate-active enzymes (CAZymes) in *Halomonas* sp. H5.

Functional analysis of carbohydrate-active enzymes using the CAZy v13 database (Figure 6) demonstrated that strain H5 not only catalyzes carbohydrate degradation but also encodes enzymes involved in the modification and synthesis of glycosidic bonds. These include glycoside hydrolases (GHs, *n* = 68), glycosyltransferases (GTs, *n* = 70), carbohydrate esterases (Ces, *n* = 7), and auxiliary activity enzymes (Aas, *n* = 6) within their respective families. Furthermore, the H5 genome harbors 22 carbohydrate-binding modules (CBMs), which enhance substrate recognition and binding by mediating enzyme–carbohydrate interactions.

##### Analysis of Secondary Metabolite Synthesis Gene Clusters

**Figure 7:**
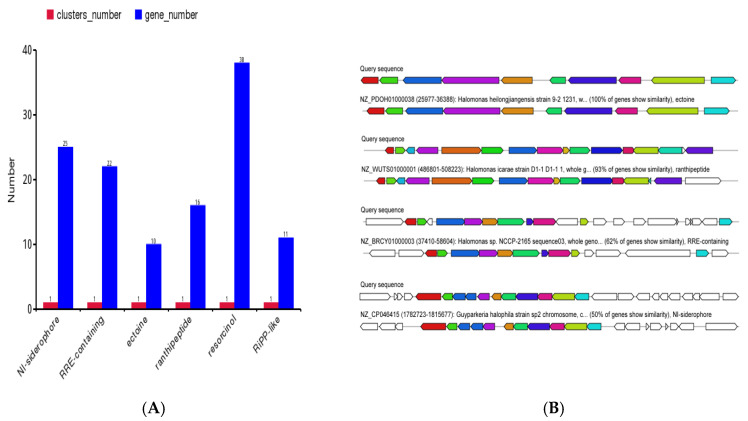
Types and quantities (**A**) and similarity analysis (**B**) of secondary metabolite gene clusters in *Halomonas* sp. H5.

The genomic analysis revealed distinct similarities between the characterized gene clusters and those of documented halophilic species. Strain H5 harbored six biosynthetic gene clusters associated with NI-siderophore (25 genes), RRE-containing peptides (22 genes), ectoine (10 genes), ranthipeptide (16 genes), resorcinol (38 genes), and RiPP-like compounds (11 genes) (Figure 7A). Specifically, the ectoine gene cluster had 100% of genes showing similarity with *Halomonas heilongjiangensis* strain 9-2 1231. The ranthipeptide gene cluster had 93% of genes showing similarity with *Halomonas icarae* strain D1-1. The RRE-containing peptides gene cluster had 62% of genes showing similarity with *Halomonas* sp. NCCP-2165. The NI-siderophore gene cluster had 50% of genes showing similarity with *Guyparkeria halophila* strain sp2 (Figure 7B). These clusters are potentially involved in plant growth promotion and salt tolerance mechanisms.

##### Analysis of VFDB Annotation Results

Comparative analysis against the VFDB database revealed that strain H5 possessed 300 annotated virulence-associated genes, representing 7.95% of its total genomic coding capacity. Gene annotation analysis identified multiple virulence-associated functional categories. These genes include genes associated with biofilm formation, such as alginate (6 genes), flagella (17 genes), and type IV pili (21 genes), genes related to immune defense, including capsule polysaccharides (10 genes), LOS oligosaccharide synthesis genes (LOS, 13 genes), and lipopolysaccharide genes (LPS, 6 genes). It also identified genes encoding antimicrobial activity mediators, notably the VAS T6SS cluster (9 genes); genes involved in siderophore synthesis/transport (SodB, 1 gene; TTSS secreted effectors, 1 gene; mycobactin, 1 gene; pyoverdine, 1 gene; FbpABC, 1 gene; HitABC, 3 genes; and CcmC, 4 genes)ge; nes encoding key enzymes for pantothenate (vitamin B5) and β-alanine biosynthesis (PanC/PanD, 2 genes); phosphate uptake/transport genes (T4SS secreted effectors, 4 genes); and nitrogen metabolism/regulation genes (urease, 4 genes; allantoin utilization, 1 gene). These virulence-associated genetic elements collectively enhance the strain’s defense against pathogens and environmental adaptability.

#### 3.1.3. Genes Associated with Salt-Tolerance and Plant Growth Promotion in H5 Genome

Functional analysis of the H5 genome revealed the presence of multiple genes involved in various salt-tolerance and plant growth-promoting functions, including compatible solute biosynthesis genes, ion transport and pH regulation genes, osmotic regulation and stress response genes, plant hormone biosynthesis genes, nutrient metabolism and transport genes, antioxidant and stress response, and microbial plant interactions (Table 1 and Table 2).

### 3.2. Effects of Halomonas sp. H5 on Salt-Tolerant Characteristics of Tomato 

**Figure 8:**
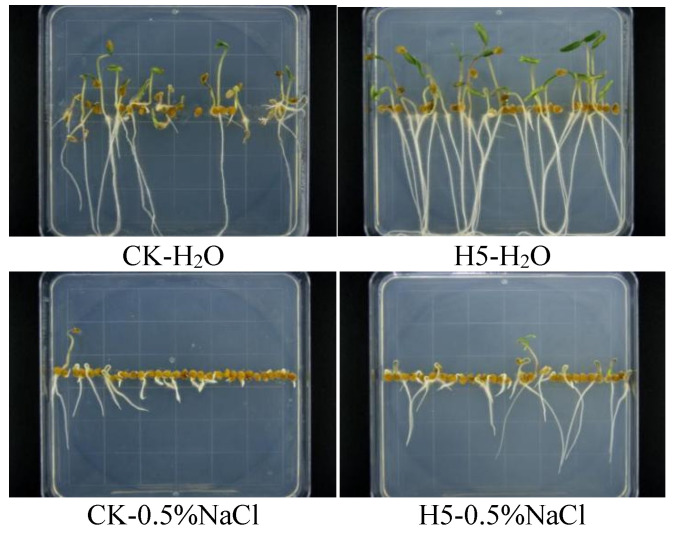
Effects of *Halomonas* sp. H5 on salt-tolerant characteristics of tomato seedlings.

Plate-based bioassays demonstrated that NaCl exerted significant inhibitory effects on tomato seed germination and seedling development. Compared to the CK treatment (0% NaCl), the H5 treatment in sterile water (0% NaCl) significantly enhanced germination rates, accompanied by increased root length and plant height (*p* < 0.05). Compared to the CK treatment (5% NaCl), the H5 treatment under moderate salt stress (0.5% NaCl) significantly promoted tomato root growth (*p* < 0.05), though no significant effects were observed on seed germination or seedling shoot development (Figure 8). The growth-promoting efficacy of H5 should be further analyzed through potted experiments under salt stress.

### 3.3. Effects of Halomonas sp. H5 on Tomato Growth, Physiological Responses, and Antioxidant Enzyme Activities Under Salt Stress

#### 3.3.1. Effects of *Halomonas* sp. H5 on Tomato Growth Under Salt Stress

**Figure 9:**
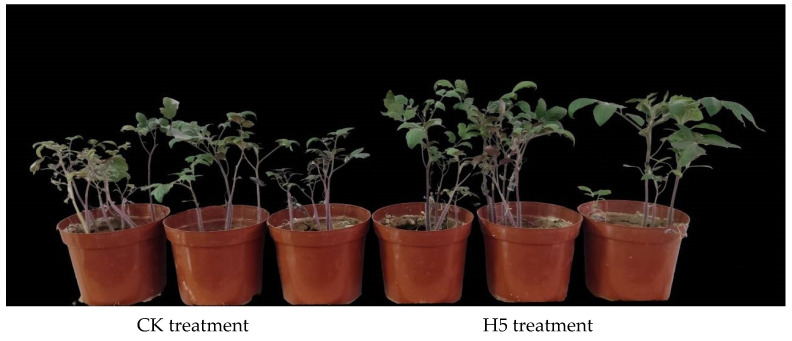
Pot experiment on the effect of *Halomonas* sp. H5 on tomato growth under salt stress.

The pot experiments demonstrated that the H5 treatment enhanced tomato growth under salt stress (Figure 9). Compared to the CK treatment, the emergence rate, plant height, and fresh weight of tomatoes significantly increased by 16.68%, 20.87%, and 40.59%, respectively (*p* < 0.05, Table 3).

#### 3.3.2. Effects of *Halomonas* sp. H5 on Tomato Physiology Responses Under Salt Stress

**Figure 10:**
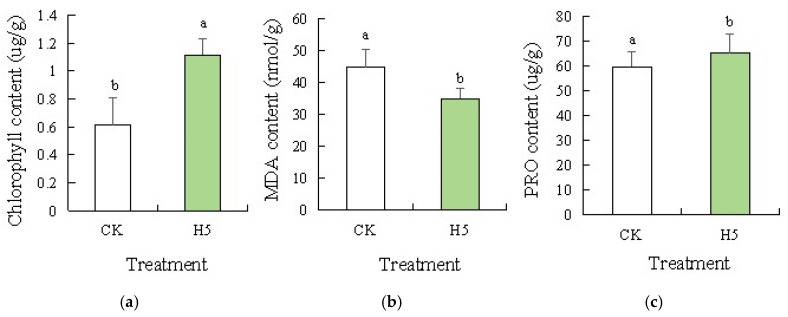
Effects of *Halomonas* sp. H5 on tomato physiology responses in tomato leaves under salt stress. Different lowercase letters in the same column indicate a significant difference at the *p* < 0.05 level. The same as below. (**a**) chlorophyll content, (**b**) MDA content, (**c**) PRO content.

Analysis of physiological parameters, including chlorophyll content (Figure 10a), MDA content (Figure 10b), and PRO content (Figure 10c) in tomato leaves under salt stress, revealed distinct trends. Compared to the CK treatment, chlorophyll and proline content significantly increased by 81.60% (*p* < 0.05) and 9.47%, respectively. Malondialdehyde content significantly decreased by 21.92% (*p* < 0.05) in the H5 treatment.

#### 3.3.3. Effects of *Halomonas* sp. H5 on Tomato Antioxidant Enzyme Activities Under Salt Stress

**Figure 11:**
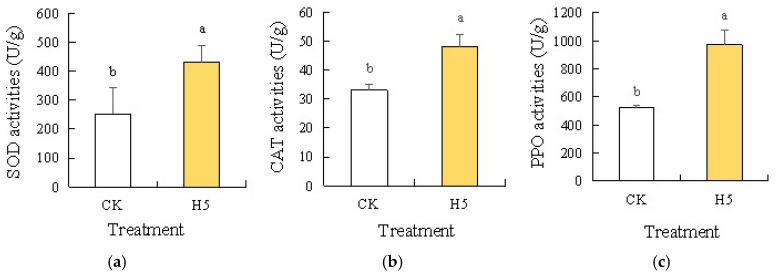
Effects of *Halomonas* sp. H5 on antioxidant enzyme activities in tomato leaves under salt stress. Different lowercase letters in the same column indicate a significant difference at the *p* < 0.05 level. The same as below. (**a**) SOD activities, (**b**) CAT activities, (**c**) PPO activities.

Analysis of antioxidant enzyme activities in tomato leaves under salt stress revealed significant increases in SOD (Figure 11a), CAT (Figure 11b), and PPO (Figure 11c) following H5 treatment. Compared with the CK treatment, the H5 treatment significantly increased by 70.17% (SOD), 45.42% (CAT), and 86.45% (PPO) in enzymatic activities (*p* < 0.05).

### 3.4. Effects of Halomonas sp. H5 on the Physicochemical Properties and Microbial Diversity of Tomato Rhizosphere Soil Under Salt Stress

#### 3.4.1. Effects of *Halomonas* sp. H5 on Salinity-Alkalinity Parameters and Nutrient Contents of the Tomato Rhizosphere Soil Under Salt Stress

As shown in Table 4, the H5 treatment significantly altered saline-alkaline and nutrient parameters in tomato rhizosphere soil. Compared to the CK treatment, the pH of the rhizosphere soil in the H5 treatment showed no significant difference. However, total salt content and electrical conductivity (EC) of the H5 treatment decreased by 9.33% and 8.09%, respectively (*p* < 0.05). For soil nutrient content, available nitrogen content significantly decreased by 31.30% (*p* < 0.05), whereas organic matter and total nitrogen content significantly increased by 11.19% and 10.81%, respectively (*p* < 0.05).

#### 3.4.2. Effects of *Halomonas* sp. H5 on Microbial Community Diversity Indices in Tomato Rhizosphere Soil Under Salt Stress

**Figure 12:**
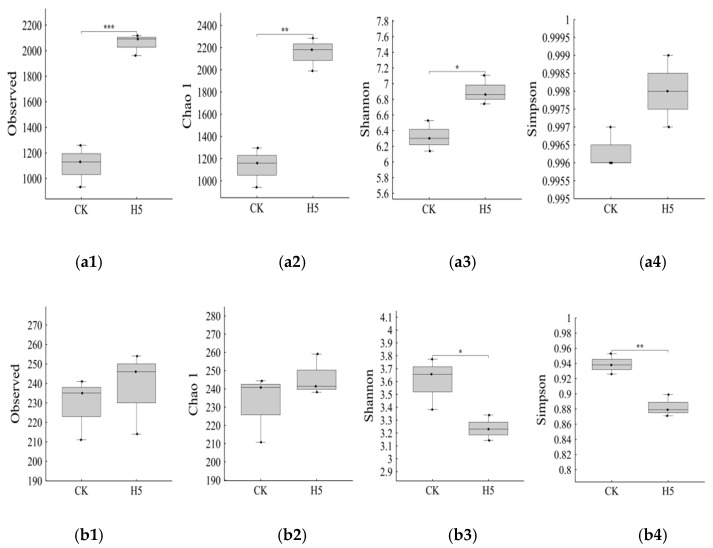
Effects of *Halomonas* sp. H5 on bacterial and fungal community diversity indices in tomato rhizosphere soil under salt stress. (**a1**) observed OTU of bacteria, (**a2**) Chao 1 diversity indices of bacteria, (**a3**) Shannon diversity indices of bacteria, (**a4**) Simpson diversity indices of bacteria, (**b1**) observed OTU of fungi, (**b2**) Chao 1 diversity indices of fungi, (**b3**) Shannon diversity indices of fungi, (**b4**) Simpson diversity indices of fungi. *: *p* < 0.05, **: *p* < 0.01, ***: *p* < 0.001.

The H5 treatment significantly influenced diversity indices of bacterial and fungal communities in tomato rhizosphere soil. Compared to the CK treatment, the H5 treatment significantly increased the number of observed bacterial OTUs (Figure 12(a1)) by 85.82% (*p* < 0.01). Similarly, the Chao1 abundance index (Figure 12(a2)) significantly increased by 89.91% (*p* < 0.01), and the Shannon diversity index (Figure 12(a3)) increased by 9.16% (*p* < 0.05). For fungal communities, the H5 treatment showed no significant differences from the CK treatment in either observed OTUs (Figure 12(b1)) or the Chao1 (Figure 12(b2)) abundance index. However, the Shannon diversity index (Figure 12(b3)) decreased by 10.18% (*p* < 0.05).

#### 3.4.3. Effects of *Halomonas* sp. H5 on Phylum-Level Composition of Bacterial and Fungal Communities in Tomato Rhizosphere Soil Under Salt Stress

**Figure 13:**
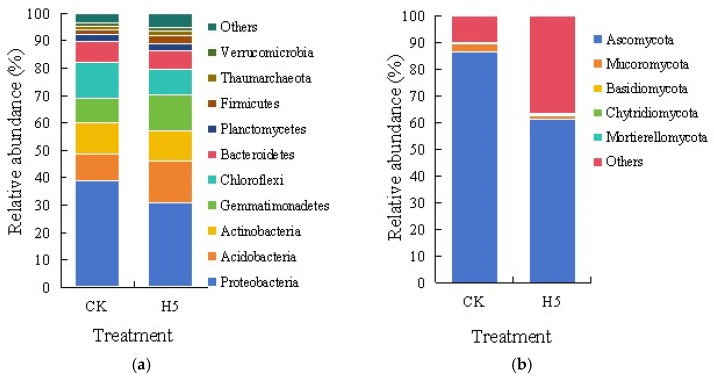
Effects of *Halomonas* sp. H5 on bacterial (**a**) and fungal (**b**) communities at the phylum level in tomato rhizosphere soil under salt stress.

As delineated in Figure 13a, Proteobacteria constituted the dominant phylum in soil bacterial communities, accounting for over 30% of the total sequences. However, the H5 treatment significantly increased their relative abundance by 20.23% compared to the CK treatment (*p* < 0.05). Chloroflexi and Actinobacteria emerged as the subsequent predominant phyla, with Chloroflexi showing a marked decrease of 27.43% under the H5 treatment (*p* < 0.05), while Actinobacteria exhibited no statistically significant variation. In contrast, Acidobacteria and Gemmatimonadetes demonstrated substantial enrichment, increasing by 55.32% and 44.43%, respectively, in the H5 treatment (*p* < 0.05). Other phyla collectively represented minor components of the soil bacterial communities.

As shown in Figure 13b, Ascomycota constituted the dominant phylum in soil fungal communities, with relative abundances of 86.40% and 61.29% in the CK and H5 treatments, respectively. The H5 treatment significantly decreased its relative abundance by 29.06% compared to the CK treatment (*p* < 0.05). Mucoromycota emerged as the second most abundant phylum, exhibiting relative abundances of 3.23% (CK) and 1.25% (H5), corresponding to a 61.30% decrease under the H5 treatment (*p* < 0.05). Other phyla collectively represented minor components of the soil fungal communities.

#### 3.4.4. Effects of *Halomonas* sp. H5 on Genera-Level Composition of Bacterial and Fungal Communities in Tomato Rhizosphere Soil Under Salt Stress

**Figure 14:**
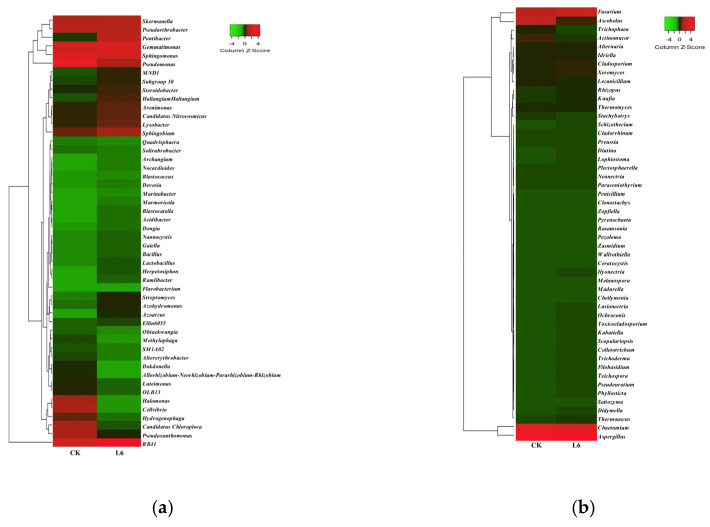
Effects of *Halomonas* sp. H5 on bacterial (**a**) and fungal (**b**) communities at the genus level in tomato rhizosphere soil under salt stress.

Through ASV (amplicon sequence variant) annotation analysis, the top 50 most abundant bacterial and fungal taxa taxonomically assigned to the genus level were identified (Figure 14). At the genus level for bacteria (Figure 14a), the dominant bacterial genera were those unclassified under the phyla Chloroflexi, Acidobacteria, and Gemmatimonadetes, with relative abundances accounting for 14.25% and 15.16% of the total abundance in the CK and H5 treatments, respectively. Furthermore, the H5 treatment significantly enriched identifiable bacterial genera, including RB41 (Acidobacteria), *Gemmatimonas*, *Sphingobium*, *Lysobacter*, *Pontibacter*, Subgroup 10 (Acidobacteria), MND1 (Proteobacteria), *Haliangium*, *Azohydromonas*, *Streptomyces*, *Azoarcus*, *Ramlibacter*, and *Herpetosiphon*. Their relative abundances significantly increased by 76.47%, 24.92%, 13.66%, 9.87%, 134.89%, 11.43%, 14.66%, 44.03%, 29.91%, 51.95%, 95.65%, 32.00%, and 39.28%, respectively (*p* < 0.05).

At the genus level for fungi (Figure 14b), the dominant fungal genera were *Aspergillus*, *Chaetomium*, *Ascobolus*, and *Fusarium*, with relative abundances accounting for 52.83% and 36.57% of the total abundance in the CK and H5 treatments, respectively. However, the remaining top 50 fungal genera showed varying degrees of reduction. Particularly marked declines were observed in *Ascobolus*, *Actinomucor*, *Trichophaea*, *Cheilymenia*, *Madurella*, *Zopfiella*, *Clonostachys*, *Penicillium*, *Pezoloma*, *Rasamsonia*, *Pyrenochaeta*, and *Zasmidium*. Their relative abundances significantly decreased by 80.57%, 82.45%, 89.48%, 91.51%, 100.00%, 100.00%, 89.66%, 100.00%, 100.00%, 100.00%, 100.00%, and 82.35%, respectively (*p* < 0.05).

#### 3.4.5. Effects of *Halomonas* sp. H5 on the Functional Diversity of Bacteria and Fungi of Tomato Rhizosphere Soil Under Salt Stress

**Figure 15:**
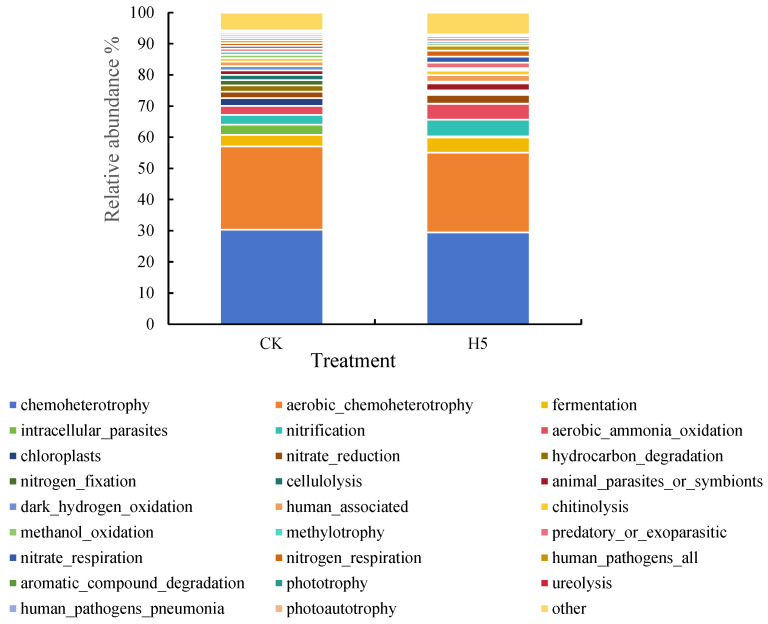
Effects of *Halomonas* sp. H5 on bacterial functions in tomato rhizosphere soil under salt stress.

The potential ecological functions of rhizosphere soil bacterial communities in the H5 treatment were predicted and annotated using the FAPROTAX database (Figure 15). Functional annotation revealed 59 distinct functional groups, with chemoheterotrophy and aerobic chemoheterotrophy exhibiting the highest relative abundance among the predicted metabolic categories (29.45–30.29% and 25.61–26.77%, respectively). Subsequent analysis of bacterial functional taxa with a relative abundance ≥0.5% revealed that the H5 treatment significantly increased carbon-associated metabolic processes. Specifically, fermentation, chitinolysis, and phototrophy significantly increased by 32.37%, 26.22%, and 14.99%, respectively (*p* < 0.05). The treatment also significantly increased nitrogen-cycling functions, including nitrification, aerobic ammonia oxidation, nitrate reduction, nitrate respiration, nitrogen respiration, and ureolysis, by 71.37%, 73.24%, 30.15%, 120.30%, 120.30%, and 30.39%, respectively (*p* < 0.05). Additionally, the H5 treatment significantly increased the relative abundance of animal parasites or symbionts by 56.72% and predatory or parasitic functions by 77.79%. Notably, some bacterial groups associated with human pathogens also showed significant increases, but these functional categories constituted only a minor proportion of the total community.

**Figure 16:**
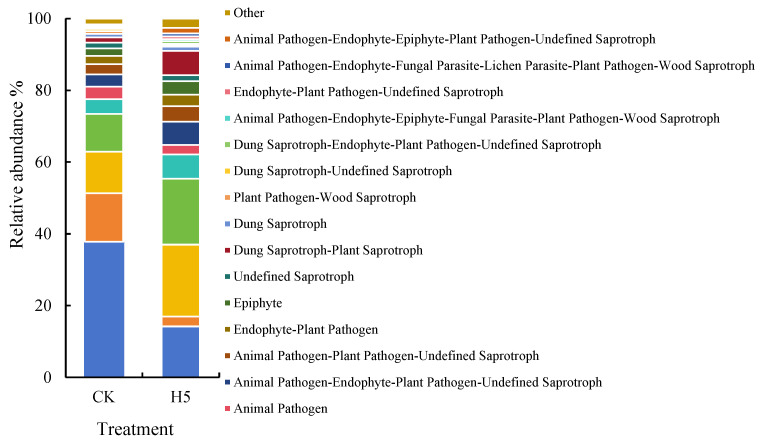
Effects of *Halomonas* sp. H5 on fungal functions in tomato rhizosphere soil under salt stress.

FUNGuild database analysis of potential fungal ecological functions (Figure 16) revealed that the dominant fungal functions were Dung Saprotroph and Wood Saprotroph; Dung Saprotroph and Ectomycorrhizal; Plant Pathogen and Undefined Saprotroph; and Animal Pathogen, Endophyte, Lichen Parasite, Plant Pathogen, Soil Saprotroph, and Wood Saprotroph (37.79–14.19%, 13.56–2.76%, 11.54–20.04%, and 10.51–18.34%, respectively) (*p* < 0.05). Compared to the CK treatment, the H5 treatment significantly decreased the relative abundances of Dung Saprotroph–Wood Saprotroph and Dung Saprotroph–Ectomycorrhizal functions by 62.45% and 79.67%, respectively (*p* < 0.05). Conversely, the other two fungal functions associated with pathogenic and saprophytic increased significantly by 73.59% and 74.43%, respectively. (*p* < 0.05).

## 4. Discussion

Xinjiang, China’s predominant tomato cultivation region, faces critical agricultural challenges due to water scarcity, persistent aridity, and expansive saline-alkalized soils. These abiotic constraints severely limit tomato yields and compromise fruit quality parameters [38]. To achieve sustainable intensification of tomato production, developing ecological approaches for soil salinity mitigation is imperative. The application of PGPR represents a promising bioaugmentation strategy. This bio-restoration approach not only improves crop productivity but also fosters soil health restoration under salt stress. *Halomonas* sp., a ubiquitous halotolerant genus in saline-alkaline environments, demonstrates significant potential for development as microbial fertilizers in agricultural applications, offering valuable strain resources for saline-alkali soil amelioration [39,40]. This study conducted a comprehensive functional annotation of the whole genome of *Halomonas* sp. H5 to elucidate genetic determinants underlying its halotolerance, plant growth-promoting traits, and biocontrol capabilities. Functional annotation across multiple genomic databases revealed that the majority of genes in *Halomonas* genomes are predominantly associated with essential biological processes, including cellular maintenance, nutrient assimilation, environmental stress adaptation, and phytostimulation in plant hosts [41]. These gene clusters are enriched with functional elements associated with diverse metabolic pathways. Particularly, genomic analysis identified secretion systems responsible for bioactive metabolites that contribute to cellular homeostasis, enhance salt stress adaptation, and mitigate stress-induced damage, thereby promoting the growth and development of host plants [42,43,44,45].

Genomic analysis of *Halomonas* sp. H5 revealed genes associated with bacterial motility mechanisms, including chemotaxis systems and flagellar assembly [46]. These genetic determinants enable the strain to sense environmental cues, thereby facilitating active navigation away from high-salinity stress through directed motility responses. Strain H5 mitigated bacterial damage under salt stress by regulating osmotic homeostasis via prokaryotic quorum-sensing mechanisms and enhancing biofilm formation [47]. Functional genes in environmental signal transduction systems, such as ABC transporters, two-component systems, phosphatidylinositol signaling systems, HIF-1 signaling pathways, and plant MAPK signaling pathways, maintained relative intracellular stability by regulating osmotic pressure inside and outside the cells, promoting K^+^ uptake, reducing intracellular Na^+^ concentration, and inducing biosynthesis of compatible solutes and activating antioxidant enzymes. This coordinated regulation not only mitigates salt-induced cellular damage by preserving membrane integrity and protein functionality, but also significantly improves ionic equilibrium under hypersaline conditions. The synergistic action of these molecular mechanisms substantially enhanced the strain’s adaptive capacity in saline environments through both osmotic adjustment and oxidative stress mitigation [48].

The strain H5 possessed an extensive metabolic gene repertoire, with the majority of its genetic determinants involved in critical pathways directly associated with plant stress resistance and growth promotion. These functional modules encompass the biosynthesis of amino acids, cofactors, vitamins, polysaccharides, carbon/nitrogen assimilation processes, and secondary metabolite production [49]. Notably, under environmental stress conditions (e.g., drought and salinity), these metabolic products act as signaling molecules that regulate osmotic homeostasis in plants, thereby enhancing their resilience to abiotic stresses. *Halomonas* species secreted osmoprotectants and compatible solutes (e.g., proline, ectoine, and glycine), which enhance plant salt tolerance and stimulate growth. Under osmotic stress induced by high salinity or drought, these solutes mediate osmotic equilibrium across microbial cell membranes, thus preventing cellular dehydration and ensuring physiological homeostasis [50]. The NI-siderophore biosynthesis gene cluster harbored in *Halomonas* sp. H5 facilitates plant growth through multiple mechanisms. This genetic determinant facilitates sequestration of environmental iron ions, reduces competitive Na^+^ uptake in plants, recruits beneficial rhizosphere microbiota through chemotactic signaling, and enhances iron bioavailability for plant metabolic processes [51]. The tryptophan metabolic pathway in the strain H5 mediated the biosynthesis of IAA through sequential enzymatic conversions. qRT-PCR showed the transcript levels of amiE and aldH genes, which had been predicted to encode indole-3-acetamide hydrolase and indole-3-acetaldehyde dehydrogenase, to be significantly upregulated in response to tryptophan [52]. Furthermore, *Halomonas* sp. H5 possessed various secondary metabolic gene clusters and virulence factor genes associated with the biosynthesis of bioactive compounds, including antimicrobial agents (e.g., resorcinol) and antioxidant peptides. These bioactive substances enhance protein stability in *Halomonas*, endowing these bacteria with superior functional characteristics for agricultural biotechnology applications, particularly in stress resistance, pathogen defense, and growth promotion.

The pot experiment demonstrated that *Halomonas* sp. inoculation significantly enhanced tomato seedling emergence and growth under moderate salt stress conditions. The H5 treatment induced notable physiological improvements, including increased chlorophyll content and proline accumulation in seedling leaves, along with a marked reduction in MDA content. Furthermore, the inoculated plants exhibited significantly enhanced antioxidant enzyme activities of SOD, CAT, and PPO. These physiological modifications collectively improved the osmotic adjustment capacity and strengthened the antioxidant defense system in tomato plants, thereby enhancing their adaptability and stress resistance under salt stress. The study conducted by Masmoudi et al. demonstrated that *Halomonas* strains isolated from saltern ecosystems exhibited dual tolerance mechanisms to hypersaline and heavy metal stress concentrations, and the production of biosurfactants, exopolysaccharides, and extracellular hydrolytic enzymes, as well as capabilities for biofilm formation and secretion of plant growth-promoting compounds [53]. Additionally, studies by Ouali K O et al. indicated that *Halomonas* sp. exhibited better plant growth-promoting effects under elevated salinity conditions [54]. During salt stress adaptation, *Halomonas* sp. employed a putative Ca^2+^-transport-related protein (orf03282) that modulates intracellular Ca^2+^ homeostasis, thereby coordinating osmotic balance regulation and enhancing cellular salinity tolerance [55]. Concurrently, this bacterium synthesizes phenolic compounds and other antifungal agents [54]. These functions induced plants to strengthen their antioxidant defense capabilities and promoted the absorption of nutrients.

Furthermore, salt stress exerts adverse effects on soil health (e.g., soil nutrient availability and microbial diversity), ultimately leading to soil degradation. The research indicates that PGPR capable of tolerating high salinity and high pH levels not only demonstrate remarkable adaptability to saline-alkali soil environments, but also enhance soil fertility through secretion of diverse bioactive metabolites. These microorganisms simultaneously stimulate crop growth to improve nutrient uptake efficiency. Consequently, this dual mechanism induces indirect modifications to soil pH and salinity levels while optimizing the biomass and community structure of rhizosphere microorganisms. Such ecological improvements further ameliorate the rhizosphere microenvironment, and enhance photosynthetic efficiency and stress resistance in plants, ultimately achieving the dual objectives of soil rehabilitation and yield enhancement [56,57]. This study revealed that without exogenous fertilizer supplementation, the H5 treatment effectively reduced rhizosphere soil salinity through multiple mechanisms. Firstly, *Halomonas* sp. secreted extracellular polymers and biofilm matrices that adsorbed and sequestered excess sodium and chloride ions, reducing their bioavailability in the soil solution. Secondly, *Halomonas* inoculation stimulated tomato growth and root development, enhancing water uptake and accelerating leaching of soluble salts from the root zone. Additionally, *Halomonas* inoculation promoted the establishment of salt-tolerant PGPR populations in the tomato rhizosphere. These microbial activities facilitated ion exchange processes, immobilizing salts within microbial biomass or modulating soil ion homeostasis, thereby further reducing the bioavailable salt concentration [58]. The soil nutrient analysis revealed a decreasing trend in the contents of available nitrogen and available potassium, with a significant decrease in available nitrogen, which might be associated with plant growth requirements. The increase in available phosphorus content in the soil was probably due to the phosphorus-solubilizing capacity of *Halomonas* sp. The observed increase in organic matter and total nitrogen content might be attributed to the combined effects of microbial inoculants and root-exuded metabolites, which potentially enhanced soil nutrient status through biochemical interactions in the rhizosphere environment.

Soil microorganisms regulate the balance of microbial diversity in agroecosystems and play a crucial role in promoting plant growth. Studies on the rhizosphere microbial community of cotton in coastal saline-alkali soils had shown that excessive salinity reduced soil stability, altered microbial community structure and diversity, and ultimately led to a decrease in beneficial microbial populations [59]. Research had demonstrated that the inoculation of PGPR under salt stress could influence the structure and functional diversity of rhizosphere microbial communities. Through selective enrichment of beneficial bacterial taxa, PGPR-mediated restructuring optimizes proportions of various microbes in the soil, thereby indirectly amplifying community biodiversity and conferring enhanced salt stress resilience in plants [60,61]. Our study demonstrated that *Halomonas salifodinae* H5 significantly enhanced rhizospheric bacterial and fungal species richness indices and bacterial diversity indices and reduced fungal diversity indices in tomato by modulating α-diversity indices.

Microbial community analysis revealed that *Halomonas salifodinae* H5 treatment had notable effects on the relative abundance of bacterial phyla. The H5 treatment significantly decreased the relative abundances of Proteobacteria and Chloroflexi, respectively, while significantly enhancing the relative abundances of Acidobacteria, Gemmatimonadetes, and Firmicutes compared to CK treatment. Proteobacteria, despite their indispensable roles in biogeochemical cycling of carbon and nitrogen, exhibit reduced relative abundance in disease-suppressive soils. This phylum encompasses numerous pathogenic bacterial taxa [62]. The application of *Halomonas salifodinae* H5 demonstrates potential to mitigate phytopathogen risks, possibly through competitive exclusion or modulation of microbial community dynamics. The significant enrichment of Acidobacteria in soil ecosystems promotes the decomposition of organic matter such as plant residues and humus. Notably, some specific Acidobacteria species secrete acidic metabolites capable of solubilizing inorganic phosphates, thereby enhancing phosphorus bioavailability and promoting plant growth through improved nutrient acquisition. The Gemmatimonadetes phylum demonstrates ecologically significant functional roles, particularly in nitrogen fixation processes, and exhibits antimicrobial activity against competing microbial species. Furthermore, the Firmicutes phylum exhibits versatile metabolic capabilities contributing to critical ecosystem services, including but not limited to atmospheric nitrogen assimilation, complex organic compound mineralization, environmental stress adaptation mechanisms, biocontrol potential through microbial antagonism, and bioremediation applications for pollutant degradation [63,64,65]. At the genus level, *Halomonas salifodinae* H5 selectively enriched some plant growth-promoting rhizobacteria, including *Sphingobium*, *Lysobacter*, *Pontibacter*, *Azohydromonas*, *Streptomyces*, *Azoarcus*, and *Ramlibacter* and so on. This consortium established a complex symbiotic network through synergistic metabolic interactions to improve plant salt tolerance and promote growth [66,67,68,69,70,71,72]. FAPROTAX-based functional annotation revealed that *Halomonas* sp. inoculation significantly enriched soil taxa associated with biogeochemical cycling processes, thereby promoting carbon and nitrogen metabolic functions, potentially contributing to improved soil redox buffering capacity under saline-alkaline conditions.

Fungal community analysis revealed Ascomycota (the dominant phylum) exhibiting a 29.06% reduction in relative abundance. Concurrently, we observed pronounced enrichment of Chytridiomycota and Mortierellomycota with low relative abundance. Notably, most species of Chytridiomycota demonstrate specialized enzymatic capabilities in recalcitrant polymer decomposition, particularly cellulose hydrolysis and chitinase activity. Mortierellomycota are functionally associated with soil organic matter transformation through lipid biosynthesis and carbon catabolic pathways [73,74]. Functional annotation using FUNGuild demonstrated that *Halomonas* seed treatment significantly enriched fungal taxa with dual functional traits of pathogenicity and saprophytism. However, *Alternaria*, which is a causative agent of early blight and black spot disease in tomatoes, and *Fusarium*, which causes tomato vascular wilt and root rot syndrome, exhibited significant reductions in relative abundance by 15.14% and 9.76%, respectively, with H5 treatment (*p* < 0.05).

The genome-wide interrogation of stress resistance mechanisms and plant growth-promoting traits in *Halomonas* sp. H5, coupled with its demonstrated effects on tomato growth physiology and rhizosphere microecology under salt stress, has provided critical insights for harnessing *Halomonas* sp. in agricultural biotechnology. These findings could establish a foundation for developing microbial-based strategies to enhance crop salt tolerance while maintaining soil health. Future investigations will focus on the construction of microbial synthesis or integration *Halomonas* sp. H5 with other salt-alkali remediation technologies. This multipronged strategy aims to develop synergistic bioaugmentation platforms that concurrently address plant stress adaptation and soil ecosystem sustainability in saline environments.

## 5. Conclusions

PGPR represent an excellent microbial resource with significant potential to enhance plant adaptability in saline environments. In this study, we isolated a halotolerant strain, *Halomonas* sp. H5, from saline-alkali soil. Whole-genome sequencing revealed a diverse array of functional genes associated with plant growth promotion and salt stress tolerance. The genome encodes key metabolic pathways involved in amino acid metabolism, osmoregulation, biofilm formation, quorum sensing, indole-3-acetic acid (IAA) biosynthesis, polysaccharide production, nitrogen fixation, phosphate solubilization, and siderophore biosynthesis, all of which contribute to host crop growth facilitation. Additionally, multiple salt-tolerance-related genes were identified, including those responsible for compatible solute biosynthesis, ion transport, pH homeostasis, osmotic regulation, and stress response. Pot experiments demonstrated that *Halomonas* sp. H5 significantly alleviates abiotic stress, enhances tomato growth and disease resistance, and improves soil microecological conditions. Our research provides great potential for *Halomonas* as salt-tolerant and growth-promoting bacteria in the remediation and sustainable utilization of saline land.

## Figures and Tables

**Table 1:** Genes related to salt tolerance in *Halomonas* sp. H5.

Gene Functions		Ko_id	Ko_Name	Ko_Defi	Ko_EC
Compatible solute biosynthesis genes	The ectoine biosynthesis	K05520	pfpI	protease I	3.5.1.124
		K01496	hisI	phosphoribosyl-AMP cyclohydrolase	3.5.4.19
		K09823	zur	Fur family transcriptional regulator, zinc uptake regulator	-
		K06720	ectC	L-ectoine synthase	4.2.1.108
		K00836	ectB, dat	diaminobutyrate-2-oxoglutarate transaminase	2.6.1.76
		K06718	ectA	L-2,4-diaminobutyric acid acetyltransferase	2.3.1.178
	Glycine betaine biosynthesis and transport	K06720	ectC	L-ectoine synthase	4.2.1.108
		K00836	ectB, dat	diaminobutyrate-2-oxoglutarate transaminase	2.6.1.76
		K06718	ectA	L-2,4-diaminobutyric acid acetyltransferase	2.3.1.178
		K01696	trpB	tryptophan synthase beta chain	4.2.1.20
		K06147	ABCB-BAC	ATP-binding cassette, subfamily B, bacterial	-
	Proline and glutamate metabolism	K01915	glnA, GLUL	glutamine synthetase	6.3.1.2
		K01647	CS, gltA	citrate synthase	2.3.3.1
		K13821	putA	RHH-type transcriptional regulator, proline utilization regulon repressor/proline dehydrogenase/delta 1-pyrroline-5-carboxylate dehydrogenase	1.5.5.2 1.2.1.88
Ion transport and pH regulation genes	K^+^/H^+^ antiporter	K05559, K05560, K05561, K05562, K05563, K05564	phaA, phaC, phaD, phaE, phaF, phaG	multicomponent K^+^:H^+^ antiporter subunit A	
	Na^+^/H^+^ antiporter	K05565, K05566, K05567, K05568, K05569, K05570, K05571	mnhA(mrpA), mnhB(mrpB), mnhC(mrpC), mnhD(mrpD), mnhE(mrpE), mnhF(mrpF), mnhG(mrpG)	multicomponent Na^+^:H^+^ antiporter subunit A (B, C, D, E, F, and G)	-
		K03307	TC.SSS	Solute-Na^+^ symporter, SSS family	-
		K03453	TC.BASS	bile acid-Na^+^ symporter, BASS family	-
		K03312	gltS	glutamate-Na^+^ symporter, ESS family	-
		K03308	TC.NSS	Neurotransmitter-Na^+^ symporter, NSS family	-
		K03315	nhaC	Na^+^:H^+^ antiporter, NhaC family	-
		K00346, K003467, K00348, K00349, K00350, K00351	nqrA, nqrB, nqrC, nqrD, nqrE, nqrF	Na^+^-transporting NADH-ubiquinone oxidoreductase subunit A (B, C, D, E, and F)	7.2.1.1
		K02117,	ATPVA(ntpA, atpA),	V/A-type H^+^/Na^+^-transporting ATPase subunit A	7.1.2.2 7.2.2.1
		K02118, K02120, K02121, K02123, K02124	ATPVB(ntpB, atpB), ATPVD(ntpD, atpD), ATPVE(ntpE, atpE), ATPVI(ntpI, atpI), ATPVK(ntpK, atpK)	V/A-type H^+^/Na^+^-transporting ATPase subunit B (D, E, I, and K)	-
		K11616	maeN	Malate-Na^+^ symporter	-
Osmotic regulation and stress response genes	Trehalose biosynthesis	K10236	thuE, lpqY	trehalose/maltose transport system substrate-binding protein	-
		K10238	thuG, sugB	trehalose/maltose transport system permease protein	-
		K10237	thuF, sugA	trehalose/maltose transport system permease protein	-

**Table 2:** Genes related to growth promotion in *Halomonas* sp. H5.

Gene Functions		Ko_id	Ko_Name	Ko_Defi	Ko_EC
Plant hormone biosynthesis genes	Indole-3-acetic acid (IAA) biosynthesis	K09472	puuC, aldH	4-(gamma-glutamylamino)butanal dehydrogenase	1.2.1.99
		K01426	E3.5.1.4, amiE	amidase	3.5.1.4
Nutrient metabolism and transport genes	Phosphate-solubilizing	K00262	E1.4.1.4, gdhA	glutamate dehydrogenase (NADP+)	1.4.1.4
	NI-siderophore biosynthesis	K02404	flhF	flagellar biosynthesis protein FlhF	-
		K03516	flhE	flagellar protein FlhE	-
		K02405	fliA, whiG	RNA polymerase sigma factor for flagellar operon FliA	-
		K16091	fecA	Fe (3+) dicitrate transport protein	-
		K02015	ABC.FEV.P	iron complex transport system permease protein	-
		K02016	ABC.FEV.S	iron complex transport system substrate-binding protein	-
		K02510	hpaI, hpcH	4-hydroxy-2-oxoheptanedioate aldolase	4.1.2.52
		K01586	lysA	diaminopimelate decarboxylase	4.1.1.20
		K03797	E3.4.21.102, prc, ctpA	carboxyl-terminal processing protease	3.4.21.102
		K01267	DNPEP	aspartyl aminopeptidase	3.4.11.21
		K06942	ychF	ribosome-binding ATPase	-
		K01056	PTH1, PTRH1, pth, spoVC	peptidyl-tRNA hydrolase, PTH1 family	3.1.1.29
		K02897	RP-L25, rplY	large subunit ribosomal protein L25	-
		K00948	PRPS, prsA	ribose-phosphate pyrophosphokinase	2.7.6.1
		K00919	ispE	4-diphosphocytidyl-2-C-methyl-D-erythritol kinase	2.7.1.148
		K02494	lolB	outer membrane lipoprotein LolB	-
		K02492	hemA	glutamyl-tRNA reductase	1.2.1.70
Antioxidant and stress response	Peroxisome	K01640	HMGCL, hmgL	hydroxymethylglutaryl-CoA lyase	4.1.3.4
		K01578	MLYCD	malonyl-CoA decarboxylase	4.1.1.9
		K03426	E3.6.1.22, NUDT12, nudC	NAD+ diphosphatase	3.6.1.22
		K00031	IDH1, IDH2, icd	isocitrate dehydrogenase	1.1.1.42
		K01897	ACSL, fadD	long-chain acyl-CoA synthetase	6.2.1.3
		K04564	SOD2	superoxide dismutase, Fe-Mn family	1.15.1.1
Microbial plant interactions	Bacterial chemotaxis	K05874	tsr	methyl-accepting chemotaxis protein I, serine sensor receptor	-
		K05875	tar	methyl-accepting chemotaxis protein II, aspartate sensor receptor	-
		K03406	mcp	methyl-accepting chemotaxis protein	-
		K02417	fliN	flagellar motor switch protein FliN/FliY	-
		K02416	fliM	flagellar motor switch protein FliM	-
		K02410	fliG	flagellar motor switch protein FliG	-
		K02556	motA	chemotaxis protein MotA	-
		K02557	motB	chemotaxis protein MotB	-
		K03407	cheA	two-component system, chemotaxis family, sensor kinase CheA	2.7.13.3
		K03408	cheW	purine-binding chemotaxis protein CheW	-
		K03776	aer	aerotaxis receptor	-
		K00575	cheR	chemotaxis protein methyltransferase CheR	2.1.1.80
		K03412	cheB	two-component system, chemotaxis family, protein-glutamate methylesterase/glutaminase	3.1.1.61 3.5.1.44
		K03413	cheY	two-component system, chemotaxis family, chemotaxis protein CheY	-
		K03414	cheZ	chemotaxis protein CheZ	-
	Lipopolysaccharide biosynthesis	K00979	kdsB	3-deoxy-manno-octulosonate cytidylyltransferase (CMP-KDO synthetase)	2.7.7.38
		K03270	kdsC	3-deoxy-D-manno-octulosonate 8-phosphate phosphatase (KDO 8-P phosphatase)	3.1.3.45
		K00912	lpxK	tetraacyldisaccharide 4’-kinase	2.7.1.130
		K00748	lpxB	lipid-A-disaccharide synthase	2.4.1.182
		K02847	waaL, rfaL	O-antigen ligase	2.4.1.-
		K03271	gmhA, lpcA	D-sedoheptulose 7-phosphate isomerase	5.3.1.28
		K03273	gmhB	D-glycero-D-manno-heptose 1,7-bisphosphate phosphatase	3.1.3.82 3.1.3.83
		K03272	gmhC, hldE, waaE, rfaE	D-beta-D-heptose 7-phosphate kinase/D-beta-D-heptose 1-phosphate adenosyltransferase	2.7.1.167 2.7.7.70
		K02517	lpxL, htrB	Kdo2-lipid IVA lauroyltransferase	2.3.1.241 2.3.1.-
		K02527	kdtA, waaA	3-deoxy-D-manno-octulosonic-acid transferase	2.4.99.12 2.4.99.13 2.4.99.14 2.4.99.15
		K02843	waaF, rfaF	heptosyltransferase II	2.4.99.24
		K03274	gmhD, rfaD	ADP-L-glycero-D-manno-heptose 6-epimerase	5.1.3.20
		K03269	lpxH	UDP-2,3-diacylglucosamine hydrolase	3.6.1.54
		K01627	kdsA	2-dehydro-3-deoxyphosphooctonate aldolase (KDO 8-P synthase)	2.5.1.55
		K02536	lpxD	UDP-3-O-[3-hydroxymyristoyl] glucosamine N-acyltransferase	2.3.1.191
		K02535	lpxC	UDP-3-O-[3-hydroxymyristoyl] N-acetylglucosamine deacetylase	3.5.1.108
		K00677	lpxA	UDP-N-acetylglucosamine acyltransferase	2.3.1.129
		K11211	kdkA	3-deoxy-D-manno-octulosonic acid kinase	2.7.1.166
		K06041	kdsD, kpsF	arabinose-5-phosphate isomerase	5.3.1.13

**Table 3:** Effects of *Halomonas* sp. H5 on tomato growth under salt stress.

Treatment	Rate of Emergence (%)	Plant Height (cm/Plant)	Stem Diameter (cm/Plant)	Fresh Weight (g/Plant)
CK	40.00 ± 0.00 b	14.18 ± 1.56 b	0.20 ± 0.00 a	8.77 ± 1.35 b
H5	46.67 ± 6.67 a	17.14 ± 1.49 a	0.20 ± 0.00 a	12.33 ± 1.32 a

Note: Different lowercase letters in the same column indicate a significant difference at the *p* < 0.05 level. The same as below.

**Table 4:** Effects of *Halomonas* sp. H5 on the physicochemical properties in tomato rhizosphere soil under salt stress.

Treatment	Soil Salinity and Alkali Content			Soil Nutrient Content				
	pH	Total Salt (g/kg)	EC (mS/cm)	AN (mg/kg)	AP (mg/kg)	AK (mg/kg)	OM (g/kg)	TN (g/kg)
CK	8.21 ± 0.07 a	5.36 ± 0.32 a	1.36 ± 0.07 a	169.44 ± 21.10 a	13.76 ± 0.87 a	131.00 ± 6.08 a	6.88 ± 0.27 b	0.37 ± 0.05 b
H5	8.35 ± 0.04 a	4.86 ± 0.13 b	1.25 ± 0.04 b	95.92 ± 14.31 b	14.22 ± 0.25 a	121.33 ± 7.09 a	7.65± 0.27 a	0.41 ± 0.03 a

Note: Different lowercase letters in the same column indicate significant difference at *p* < 0.05 level.

## Data Availability

The raw data generated by genome sequencing can be found in the NCBI Genome as BioSample number SAMN48545772. Other data are provided within the manuscript.
